# The EGFR Signaling Pathway Is Involved in the Biliary Intraepithelial Neoplasia Associated with Liver Fluke Infection

**DOI:** 10.3390/pathogens14070620

**Published:** 2025-06-21

**Authors:** Dmitry Ponomarev, Oxana Zaparina, Anna Kovner, Elena Hadieva, Mikhail Persidskij, Maria Pakharukova

**Affiliations:** 1 Institute of Cytology and Genetics, Siberian Branch of Russian Academy of Sciences (ICG SB RAS), 10 Akad. Lavrentiev Ave., Novosibirsk 630090, Russia; zp.oksana.93@gmail.com (O.Z.); anya.kovner@gmail.com (A.K.); pmaria@yandex.ru (M.P.); 2 Clinical Hospital of the Khanty-Mansiysk Autonomus Okrug-Ugra, Khanty-Mansiysk 628013, Russia; hadievaed@okbhmao.ru (E.H.); mixajich@yandex.ru (M.P.); 3 Department of Natural Sciences, Novosibirsk State University, 2 Pirogova Str., Novosibirsk 630090, Russia

**Keywords:** H69 cholangiocytes, proliferation, migration, excretory–secretory product, *Opisthorchis felineus*, carcinogenesis, epidermal growth factor receptor (EGFR), Toll-like receptor 4 (TLR4)

## Abstract

Foodborne trematode infections are recognized as a significant risk factor for cholangiocarcinoma (CCA) in endemic regions. Infection with the liver fluke *Opisthorchis felineus* induces precursor lesions of CCA, including the biliary intraepithelial neoplasia. The mechanisms underlying liver-fluke-associated neoplasia remain poorly understood. This study aims to identify the role of EGFR and Toll-like receptor 4-associated signaling pathways in bile duct epithelial neoplasia linked to liver fluke infection in patients, animal models, and cell models. Elevated levels of EGFR and phosphorylated EGFR were observed in the bile duct epithelium of patients with cholangiocarcinoma, as well as in the bile duct epithelium of laboratory hamsters. The EGFR content correlated with the degree of bile duct epithelial neoplasia. Additionally, a significant increase in the cell proliferation and migration rates of human H69 cholangiocytes was found, whereas those of HepG2 hepatoma cells remained unaffected following the helminth excretory–secretory product (ESP) treatment. An EGFR inhibitor eliminated the enhanced cell proliferation (*p* = 0.005) and migration (*p* = 0.001) rates. Similar outcomes were achieved using Marimastat, an inhibitor of TLR-4-associated metalloproteinases. Thus, our study unveils novel avenues for exploring the mechanisms of helminth-associated carcinogenesis and for identifying key components of ESPs that mediate their mitogenic effects.

## 1. Introduction

The liver fluke *Opisthorchis felineus* (Rivolta, 1884) is one of three epidemiologically significant foodborne trematodes within the Opisthorchiidae family (class: Trematoda and phylum: Platyhelminthes). *Opisthorchis felineus* is the primary causative agent of opisthorchiasis in Russia and several Eastern European countries [1,2]. Infection occurs through the consumption of raw or undercooked *Cyprinidae fish* [2], leading to chronic inflammation, periductal fibrosis, and biliary intraepithelial neoplasia (BilIN) [3,4,5].

Biliary intraepithelial neoplasia occurs early and frequently in the opisthorchiasis pathogenesis, in which cells undergo malignant transformation. BilIN is characterized by a substandard cellular phenotype, a multilayered appearance, and the loss of cell contact with the basement membrane [6]. Closely related to *O. felineus*, other members of the Opisthorchiidae family—*O. viverrini* and *Clonorchis sinensis* [7]—are classified by the International Agency for Research on Cancer (IARC) as Group 1A biological carcinogens [8] and major risk factors for cholangiocarcinoma in endemic regions [9,10,11]. Cholangiocarcinoma (CCA) has no specific symptoms, making it difficult to diagnose. It is characterized by slowly growing tumors that metastasize to distant organs and has a poor prognosis: the five-year survival rate with surgical intervention ranges from 15 to 25% but drops to 2% in the presence of metastases [12].

The carcinogenic potential of *O. felineus* and the development of *O. felineus*-associated CCA remain poorly understood [3,13]. Liver flukes are known to promote the proliferation of bile duct epithelium cells in vitro [14,15,16,17]. Moreover, in animal models, the carcinogenic potential of *O. felineus* was comparable to that of *O. viverrini* and *C. sinensis* [4].

To date, the mechanisms underlying the development of cholangiocarcinoma associated with Opisthorchiidae family trematode infections remain elusive. Additionally, there are insufficient data on early events that can lead to precancerous lesions and, subsequently, in combination with other factors, trigger the development of cholangiocarcinoma [18]. The development of bile duct epithelial neoplasia is associated with an increased production of reactive oxygen species [19], as well as changes in the expression of p53, HER2, and EGFR [20,21,22]. Notably, increased EGFR expression is linked to early changes in cell neoplasia [23]. Nevertheless, there are still no data on molecular pathways associated with biliary neoplasia in opisthorchiasis felinea in humans.

Among the various signaling pathways, those triggered through epidermal growth factor receptors are involved in the development of epithelial cancers [24], such as non-small cell lung cancer [25]. These pathways play crucial roles in cell proliferation, differentiation, and the regulation of apoptosis [26,27]. Furthermore, some EGFR ligands are released by matrix metalloproteases (ADAM17 and TACE), which are activated by Toll-like receptor 4 (TLR4) signaling [28]. This process leads to extracellular matrix reorganization and cell migration [29].

Nevertheless, there is still no evidence on molecular signaling events involved in the development of *O. felineus*-associated cholangiocarcinoma. In particular, no data have been reported on EGFR-associated signaling pathways. Moreover, no particular regulatory events that trigger BiLiN development have been identified. Additionally, there are no data on the expression of EGFR and EGFRp in the bile duct epithelium of *O. felineus*-infected humans or laboratory animals. Notably, several in vitro studies have demonstrated the role of the EGFR pathway in the proliferation of CCA cells [30]. It is suggested that fatty acids are directly involved in the activation of this signaling pathway [30]. Additionally, both in vivo and in vitro assessments of the Trefoil factor (TFF)-associated EGFR signaling pathway in CCA samples have been conducted. The authors suggest that inhibiting TTF2 could potentially serve as a therapeutic target in invasive CCA [31].

Thus, we focused our research not only on formed tumors but also on the early events in BiliN caused by *O. felineus* infection. We hypothesized that *O. felineus* excretory–secretory products (ESPs), which include proteins, exosome-like vesicles, miRNA, and low-molecular-weight cholesterol metabolites [32], can affect human cells [14,19,33] through the activation of EGFR-dependent signaling pathways [34].

Therefore, the aim of this study was to identify the involvement of EGFR- and TLR-4-associated signaling pathways in bile duct epithelial cells during liver fluke infection-associated neoplasia in patients and animal models, as well as in a cellular model of host–parasite interactions.

## 2. Materials and Methods

### 2.1. Ethics Statement

SPF (specific pathogen-free) Syrian hamsters (*Mesocricetus auratus*) from the Animal Facility of the Institute of Cytology and Genetics, Siberian Branch of the Russian Academy of Sciences (ICG SB RAS) were used in this study. All the procedures followed EU Directive 2010/63/EU and ARRIVE guidelines (https://arriveguidelines.org/arrive-guidelines) (accessed on 20 June 2025) for animal experiments. The animals were kept according to the protocols approved by the Committee on the Ethics of Animal Experiments at the ICG SB RAS (permit number 42 on 25 May 2018). Hamsters were examined daily for signs of illness, injury, or abnormal behavior by the animal facility’s trained personnel. Food and water availability and the macroenvironment (temperature, humidity, noise, light intensity, and cleanliness) were evaluated daily. The animals were fed a standard autoclaved rodent diet, specifically ssniff^®^ R/M-H V1534 (Soest, Germany), which was approved by the specialist overseeing the SPF Animal Facility of the Institute of Cytology and Genetics. No unexpected animal deaths were registered during this study.

This study was performed in line with the principles of the Declaration of Helsinki and approved by the Medical Ethics Committee at the Clinical Hospital of the Khanty-Mansiysk (protocol #5/2023 on 26 August 2023). Written informed consent was obtained from the next of kin, who authorized the analysis of samples.

### 2.2. Metacercariae Collection and Animals

*O. felineus* metacercariae were collected from naturally infected fish (*Leuciscus idus*) caught in the Ob River near Novosibirsk (Western Siberia, Russia) and extracted accordingly [35,36]. After several washes with normal saline, metacercariae were identified under a light microscope.

Animals (male Syrian golden hamsters, aged 6 to 8 weeks) were orally infected with 100 *O. felineus* metacercariae. Euthanasia was performed after 3 months via carbon dioxide inhalation, and every effort was made to minimize the suffering of the hamsters. Liver samples for histological and immunohistochemical analyses were fixed in 10% buffered formalin (Biovitrum, Saint-Petersburg, Russia) and processed in increasing concentrations of alcohol and xylene using STP 120 (Microm, Rotherham, UK). The tissue was then embedded in paraffin, and 4 μm paraffin slides were prepared using a Microm microtome (Microm, Rotherham, UK), as described previously [37].

### 2.3. Human Samples

Human tissue samples were collected during autopsies at the Clinical Hospital of the Khanty-Mansiysk Autonomous Okrug (Russia) between 2017 and 2021. Chronic *O. felineus* infection in patients was confirmed by medical records in autopsy reports. However, the exact duration of infection, the parasitic load as indicated by the number of eggs in the stool, and whether anthelmintic treatment was administered were unknown.

This study used autopsy material from 9 patients, including 7 men and 2 women, aged 46 to 61 years.

### 2.4. Histology and Immunohistochemistry of the Liver

Immunohistochemical analysis of liver samples from laboratory animals and autopsy material from patients was performed according to the standard protocol described previously [35]. The antibodies and dilutions used in this study were as follows: [KD Validated] EGFR Rabbit pAb (1:150; cat. No. A11577, ABClonal, Wuhan, China) and Phospho-EGFR-Y869 Rabbit pAb (1:150; cat. No. A1113, ABClonal, China). The secondary antibody used was HRP Goat Anti-Rabbit IgG (H + L) (1:500; cat. No. AS014, ABClonal, China). Visualization was performed using an AxioImager A1 microscope (Zeiss, Oberkochen, Germany) with an AxioCam MRc camera (Zeiss, Germany).

### 2.5. Isolation of Excretory–Secretory Products

Adult worms were isolated from the bile ducts of Syrian golden hamsters after 3 months of infection. Excretory–secretory products (ESPs) were isolated from conditioned media from approximately 300 adult parasites over 72 h, following a previously published protocol [25,33]. Briefly, the medium was collected twice a day and centrifuged for 10 min at 4 °C and 300–500 g, then for 20 min at 4 °C and 2000 g. The supernatant was filtered through 0.45 µm and 0.22 µm filters (MF-Millipore, Merck Millipore, Burlington, MA, USA). The resulting filtrate was loaded into centrifuge concentrators (MWCO = 10 kDa, 5 kDa, and 2 kDa to isolate different ESP fractions) and centrifuged for 30 min at 3220 g and 4 °C. The concentrated medium was then dialyzed against phosphate-buffered saline (PBS) in the same concentrators to a final volume of 100–150 µL. A protease inhibitor cocktail (GE Healthcare, Chicago, IL, USA) was added to the ESP, then ESP was aliquoted and stored at −80 °C. The protein content of ESP was measured using the BCA method (Thermo Fisher Scientific, Waltham, MA, USA). *O. felineus* ESP was purified from endotoxins using a Pierce High Capacity Endotoxin Removal Resin (Thermo Scientific, Waltham, MA, USA) following the manufacturer’s recommendations.

### 2.6. Cell Cultures

The H69 cell line is an SV40-transformed human cholangiocyte epithelial cell line (kindly provided by Professor B. Sripa, Khon Kaen University, Thailand). The cells were cultured at 37 °C in an atmosphere of 5% CO_2_ in DMEM/F12 medium (Sigma-Aldrich, St. Louis, MO, USA) supplemented with 10% fetal bovine serum (FBS) (Gibco, Thermo Fisher Scientific, USA), 100 IU/mL penicillin, 100 μg/mL streptomycin, 25 μg/mL adenine, 1 μg/mL epinephrine, 5 μg/mL insulin, 0.62 μg/mL hydrocortisone, 8.3 μg/mL transferrin, 13.6 ng/mL T3 (triiodothyronine), and 10 ng/mL epidermal growth factor (EGF) (all reagents from Sigma-Aldrich, USA).

The commercially available human hepatocyte line HepG2 (provided by Dr. T.A. Schneider, Institute of Cytology and Genetics SB RAS, Novosibirsk, Russia) was used as a control cell line. HepG2 cells were cultured in DMEM/F12 medium containing 10% FBS, 100 IU/mL penicillin, and 100 μg/mL streptomycin.

### 2.7. Cell Migration and Proliferation Rate

To study the effect of the ESP of *O. felineus*, a co-cultivation method was used with ThinCert inserts (Greiner, Kremsmünster, Austria) in a 12-well plate, featuring membranes with pore diameters of 1 µm and 8 µm. Since co-cultivation using inserts with a pore diameter of 8 µm resulted in significant cell death, it was decided to use inserts with a 1 µm pore diameter.

Cell co-cultivation with worms was performed in a low-serum medium (0.1%) and in the absence of EGF. These conditions were chosen because ESP did not have any effect on H69 cell proliferation in the medium supplemented with 10% FBS and EGF. In contrast to this, after complete removal of serum (0% FBS) during co-cultivation with 6 adult worms, the number of H69 cells increased 17 fold (*p* = 0.003); however, the cells in the untreated group did not survive. Therefore, it was decided to use a low-serum medium (0.1%) for cell proliferation tests.

ESP was added at a concentration of 40 μg/mL. BSA (bovine serum albumin) (Amresco, Dallas, TX, USA) was chosen as a non-specific control at a concentration of 40 μg/mL. ESP fractions of 2–5 kDa and 5–10 kDa were added at a concentration of 4.5 μg/mL. The cells were seeded in a 12-well plate at a concentration of 20,000 cells per well for H69 and 50,000 cells per well for HepG2.

The culture was carried out for 14 days: the cells were removed, counted, and subcultured on days 5, 9, and 14. Four technical replicates were performed.

EGFR inhibitor CAS 879127-07-8 (Calbiochem, San Diego, CA, USA) was added at a concentration of 0.1 μM, and metalloproteinase inhibitor Marimastat (Cat. No. ab141276, Abcam, Cambridge, MA, USA) was added at a concentration of 200 μM. The cells were maintained for 5 days. This experiment was carried out in a medium with the addition of 1% FBS, since cells did not survive after the treatment with inhibitors in the medium with 0.1% FBS.

The cell proliferation rates were assessed using a Goryaev cell-counting chamber (hemocytometer), Axiovert 40 CFL microscope (Zeiss, Germany), and 0.4% Trypan blue dye solution (BIO-RAD, Hercules, CA, USA). Cell counting was performed on days 5, 9, and 14. To assess the cell migration rates, a wound healing test was conducted using silicone inserts (Ibidi, Gräfelfing, Germany) as described previously [38]. The test was carried out for 9 h. The cells were seeded in the chamber and cultured for 24 h until a monolayer was formed. Then, the insert was removed, and the resulting wound was imaged using an Axiovert 40 CFL inverted light microscope (Zeiss, Germany) immediately after insert removal (0 h) and after 9 h. The wound area was determined and analyzed using the AxioVision software (version 4.8), and the migration rate was calculated. The test was performed after 14 days of co-culture with 6 adult worms or after the treatment with ESP. Experiments were repeated four times.

### 2.8. Statistics

The results were analyzed using STATISTICA 7.0 (Statsoft, Tulsa, OK, USA) Prism software packages (version 10.3.0., https://www.graphpad.com) (accessed on 20 June 2025). To check the normality of the data distribution, the D’Agostino–Pearson test and the Shapiro–Wilk test were used. For normally distributed data, statistical significance was assessed using ANOVA and post-hoc Dunnett’s or Dunn’s test. For not normally distributed data, the Kruskal–Wallis test and post-hoc Dunn’s test were used.

## 3. Results

### 3.1. Liver Immunohistochemistry

**Figure 1:**
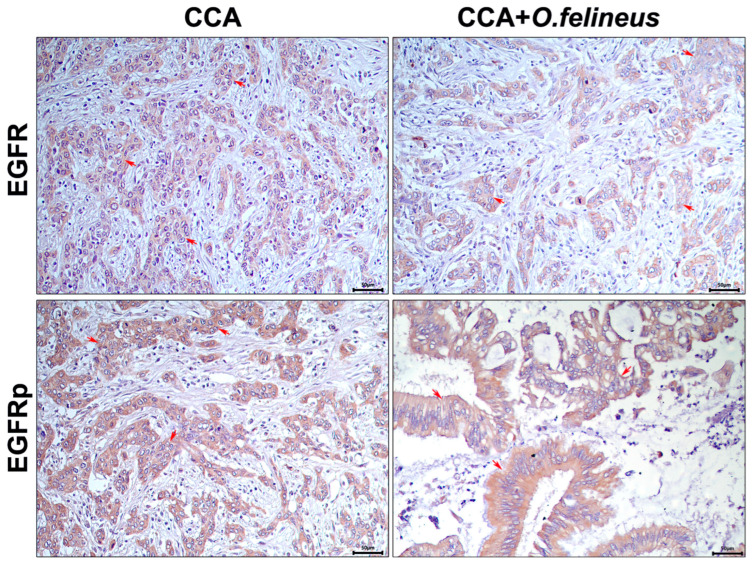
Immunohistochemistry of human liver samples (autopsy material) from patients with cholangiocarcinoma (CCA) and those with CCA associated with *Opisthorchis felineus* infection (CCA + OF); magnification ×400. EGFR: Epidermal growth factor receptor; EGFRp: Phosphorylated epidermal growth factor receptor; CCA: Cholangiocarcinoma; OF: *Opisthorchis felineus*. Specific signal areas are indicated by red arrows.

The specific staining results for the EGFR and EGFRp of the autopsy liver material from patients with cholangiocarcinoma (CCA) and those with CCA associated with *Opisthorchis felineus* infection (CCA + OF) revealed pronounced signals in the bile duct epithelium and fibrotic areas in both groups (Figure 1).

The signal was predominantly observed in the bile duct epithelium, with no significant differences in EGFR signal activation among patients with CCA only and those with CCA + OF.

**Figure 2:**
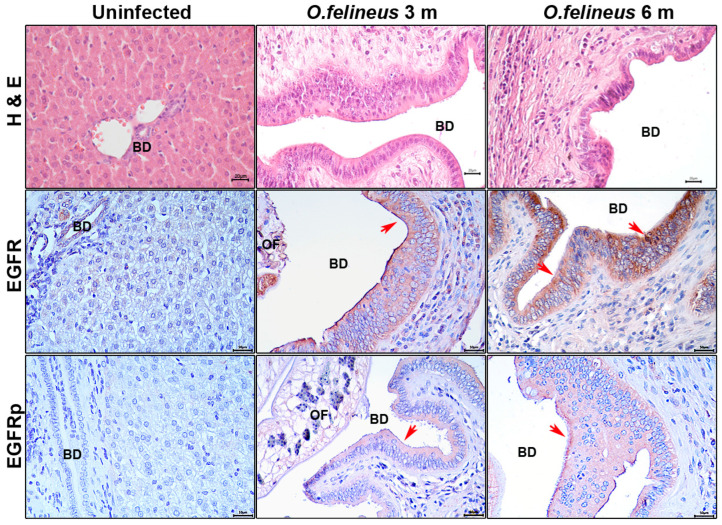
Histology and immunohistochemistry of the liver of *M. auratus* hamsters after 3 and 6 months of *Opisthorchis felineus* infection; magnification ×400. Scale: 20µm. H&E: Hematoxylin and eosin staining; BilIN: Biliary intraepithelial neoplasia; EGFR: Epidermal growth factor receptor; EGFRp: Phosphorylated epidermal growth factor receptor; BD: Bile duct; OF: *Opisthorchis felineus*. Specific signal areas are indicated by red arrows.

To investigate the EGFR content during *O. felineus* infection, we performed specific staining for EGFR and EGFRp in the livers of Syrian hamsters (*M. auratus*), a common experimental model of opisthorchiasis [36,37]. The staining was conducted at 3 and 6 months postinfection (Figure 2).

At these time points, the bile duct epithelial neoplasia was significantly pronounced in comparison to that of the uninfected animals (Figure 2), aligning with our previous data [3]. The specific EGFR signal was predominant in both the bile duct epithelium and individual cells within the periductal fibrosis area at 3 and 6 months postinfection. The specific EGFRp signal was primarily observed in the epithelium and had increased by the sixth month of infection compared to that of the uninfected animals.

Thus, both in the experimental opisthorchiasis model and in patient samples, a significant increase in EGFR content was revealed. The specific EGFR and EGFRp signals were primarily detected in the bile duct epithelium, with signal levels correlating with the epithelial neoplasia degree.

### 3.2. Proliferation and Migration Rates of Human Cholangiocytes (H69) and Human Hepatoma Cells (HepG2) in Co-Culture with Adult Opisthorchis felineus

**Figure 3:**
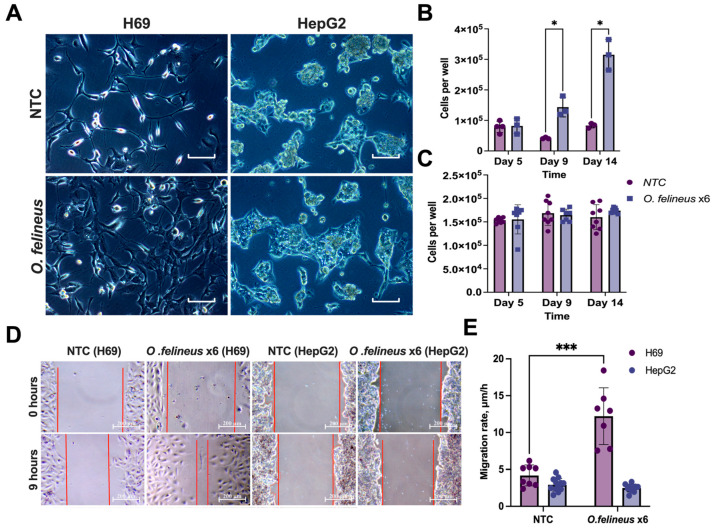
Proliferation and migration rates of H69 and HepG2 cells after co-cultivation with *O. felineus:* (**A**) Phenotypes of human cholangiocyte H69 cells and human hepatoma HepG2 cells at day 14; (**B**) Number of H69 cells after 5, 9, and 14 days of co-cultivation Scale: 100µm; (**C**) Number of HepG2 cells after 5, 9, and 14 days of co-cultivation with *Opisthorchis felineus*; (**D**) Wound healing assay. For clarity, the wound edge is marked with a red line; (**E**) Cell migration rate of H69 and HepG2 cells after 14 days of co-cultivation with adult worms. Data are presented as mean ± SD. NTCs—untreated H69/HepG2 cells; *O.felineus* x6—H69/HepG2 cells after co-cultivation with 6 helminths. Data represent the mean ± SD calculated for two visual fields per condition. * *p* < 0.05; *** *p* < 0.001 compared to untreated cells. Dunn’s post-hoc test.

The effects on the cell proliferation and migration rates during the co-cultivation of H69 cholangiocytes with adult parasites were specific to the cell line and depended on the time (Figure 3A–E).

Co-cultivation with six adult worms resulted in a significant increase in the number of H69 cells: by five times on the 9th day (*p* = 0.03) and by four times on the 14th day (*p* = 0.01) compared to the untreated cells (Figure 3B). The wound healing test revealed a two-fold increase in the H69 migration rate (*p* = 0.001) after 14 days (Figure 3E). In contrast to this, no effects were observed in HepG2 cells (Figure 3C). Representative images demonstrating an increase in the number of cells and wound healing are shown (Figure 3A,D). Therefore, subsequent experiments focused on human cholangiocytes (H69).

### 3.3. Impact of Opisthorchis felineus Excretory–Secretory Products on Cholangiocyte (H69) Proliferation and Migration

**Figure 4:**
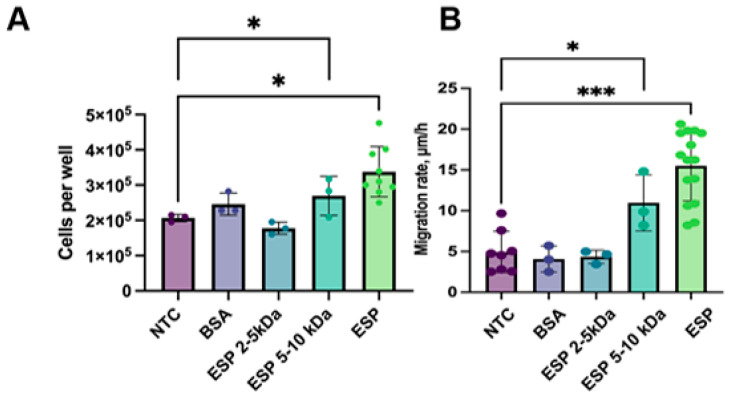
Cell number and migration rate of H69 cells after the treatment with ESP: (**A**) H69 cell count after 14 days of treatment with ESP; (**B**) Migration rate of H69 cells after 14 days of culturing with *O. felineus* ESP. NTCs—untreated H69 cells; BSA—bovine serum albumin; ESP—*O. felineus* excretory–secretory product. At least 4 biological replicates were performed. Data are presented as the mean ± SD. * *p* < 0.05; *** *p* < 0.001 compared to untreated cells (Dunn’s post-hoc test).

To identify which ESP fraction has the highest activity, we isolated the ESP and its fractions of 2–5 kDa and 5–10 kDa. After the treatment of H69 cholangiocytes with the ESP for 14 days, the number of cells increased by 1.5 times (*p* = 0.01) and by 1.2 times with the 5–10 kDa fraction (*p* = 0.04), whereas no changes were observed after the treatment with the 2–5 kDa fraction (Figure 4A).

The ESP stimulated the migration rate in H69 cholangiocytes; in particular, it was three times greater (*p* = 0.001). The 5–10 kDa ESP fraction also had an increased H69 migration rate, which was 1.3-fold greater (*p* = 0.04), but the effect was less pronounced. In contrast to this, the 2–5 kDa fraction did not significantly affect the cell migration rate (Figure 4B). Additionally, we assessed the cell proliferation and migration rates in HepG2 cells after the treatment with different ESP fractions. No significant changes were observed.

### 3.4. The Role of EGFR and Metalloproteinases in Mediating the Effects of Opisthorchis felineus Excretory–Secretory Product on Cholangiocyte (H69) Proliferation and Migration

Epidermal growth factor receptor (EGFR) ligands are synthesized as membrane protein precursors containing extracellular domains [39,40]. The cleavage of these extracellular domains by matrix metalloproteinases, such as TACE (ADAM17), releases soluble growth factors (EGFR ligands), leading to EGFR activation [41,42,43,44].

**Figure 5:**
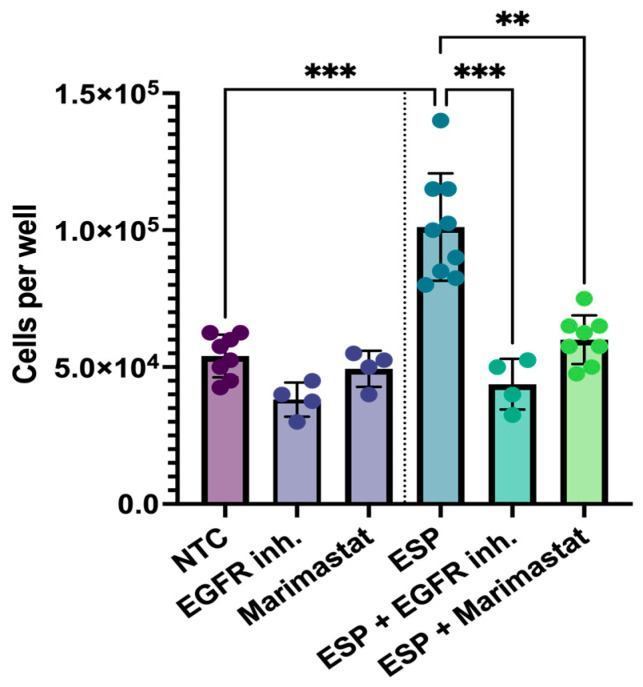
Proliferation of human cholangiocytes (H69) with Marimastat and EGFR inhibitor after treatment with the excretory–secretory product of *O. felineus*: Number of H69 cells after 5 days of ESP treatment, along with Marimastat or EGFR inhibitor. Data are presented as mean ± SD. ** *p* < 0.01; *** *p* < 0.001. Dunn’s post-hoc test; NTC: Untreated cells; EGFR inh: EGFR inhibitor; ESP: Excretory–secretory product.

To evaluate the involvement of EGFR, matrix metalloproteinases, or TLR4-associated pathways, an inhibitor test was conducted. H69 cells were cultured for 5 days with the ESP of *O. felineus*, along with either an EGFR inhibitor or the matrix metalloprotease inhibitor Marimastat. While the number of H69 cells was two times greater (*p* = 0.001) after the treatment with the ESP, the EGFR inhibitor or Marimastat eliminated this increase, maintaining cell number at levels similar to the untreated control group (NTCs) (Figure 5).

**Figure 6:**
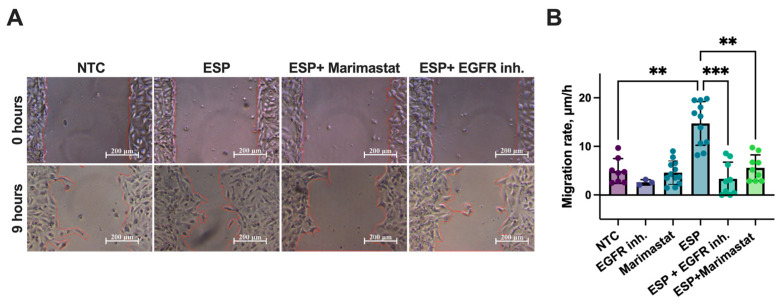
Cell migration rate of human H69 cholangiocytes treated with ESP in combination with Marimastat or EGFR inhibitor: (**A**) Wound healing test (the wound edge is marked with a red line); (**B**) H69 cell migration rate. NTC: Untreated cells; EGFR inh.: Epidermal growth factor receptor inhibitor; ESP: Excretory–secretory product. At least 4 biological replicates were performed. Data are presented as mean ± SD. ** *p* < 0.01; *** *p* < 0.001. Dunn’s post-hoc test.

A similar inhibitory effect was observed during the assessment of the cell migration rates. The migration rate of H69 cells in the ESP and EGFR inhibitor group was decreased by 4.4 fold (*p* = 0.001), while Marimastat reduced it by 2.6 fold (*p* = 0.003), compared to cells treated with the ESP alone. Notably, the cell migration rates in the groups treated with either inhibitor did not increase and remained similar to that of the untreated control group (NTCs) (Figure 6A,B).

Thus, the EGFR inhibitor and Marimastat were shown to prevent the increase in the cell proliferation and migration rates of human cholangiocytes.

## 4. Discussion

Thus, our study revealed for the first time the involvement of EGFR-associated cellular pathways in the development of BilIN in opisthorchiasis in patients, in laboratory animals, and in cell models. We focused our research on these pathways based on the following reasons. First, we were able to see the effects of the ESP on the activation of the proliferation and migration of human cholangiocytes only in the absence of EGF, reduced serum and other nutrients. This led us to think about the presence of mitogenic growth factors in ESPs. In addition, in many epithelial cancers [24], EGFR is known to be involved in cell proliferation, differentiation, and the regulation of apoptosis [25,26].

The data obtained from the autopsy material confirmed increased EGFR expression in cholangiocarcinoma human samples. However, the followed question remained: “Is the increase in EGFR expression associated with the development of cholangiocarcinoma only or does *O. felineus* infection also contribute?” To answer this, we assessed the level of EGFR expression in an experimental model of opisthorchiasis.

Thus, in a hamster model, increased EGFR expression was also demonstrated predominantly in the bile duct epithelium 3 and 6 months postinfection, which was accompanied by an increase in the degree of neoplasia. Therefore, bile duct epithelial cells are primarily exposed to the negative effects of and pathological changes during infection with liver flukes.

This raises the following questions: “What is the role of EGFR in the development of early abnormalities in the bile duct epithelium during opisthorchiasis?” and “Is the excretory-secretory product of *O. felineus* selective in its effect on the cells of the hepatobiliary system?”

**Figure 7:**
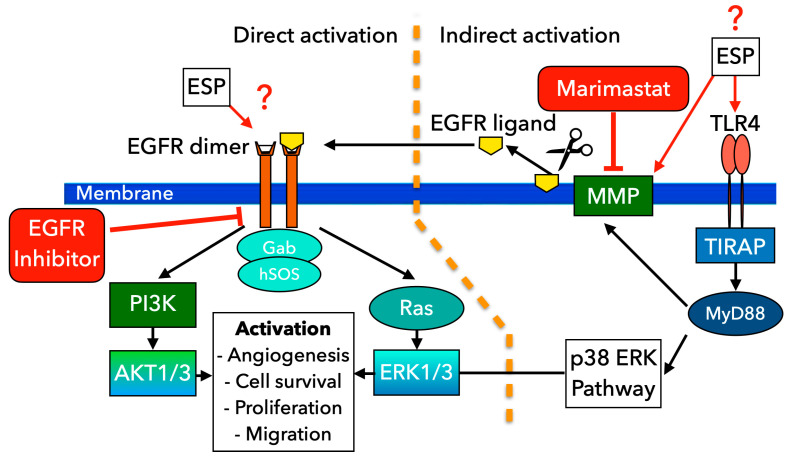
Scheme of EGFR/TLR4 signaling cascades likely involved in altering cholangiocyte proliferation and migration under the influence of the excretory–secretory product of *O. felineus*. Two main proposed pathways for EGFR activation are presented. The direct pathway of EGFR activation: *O. felineus* ESP potentially interacts with EGFR, causing activation of downstream PI3K/AKT and Ras/ERK1/3 signaling pathways followed by stimulation of angiogenesis, cell survival, cell proliferation, and migration. Alternatively, MMPs can be activated via TLR4-associated pathways, followed by the release of various EGFR ligands. These ligands then bind to EGFR. Marimastat inhibits MMP activity through direct binding, preventing the release of EGFR ligands. EGFR inhibitor can block EGFR activation, preventing its effects on cells. The scheme was prepared using GIMP 2.10 (https://www.gimp.org/) (accessed on 20 June 2025).

The selective effect of the ESP on human cholangiocytes was demonstrated in in vitro tests of cell proliferation and migration. We found that the ESP of *O. felineus* exhibits mitogenic properties, significantly enhancing both the proliferation and migration of human cholangiocyte H69 cells. This effect is selective to cholangiocytes, as the ESP of *O. felineus* did not affect HepG2 cells. Additionally, the effects of the ESP on cells were similar to those observed when cells were co-cultured with adult worms. Thus, tumor development can be accompanied by an increased expression of EGFR and phosphorylated EGFRp. Moreover, the disruption of PI3K/Akt/mTOR and RAS/RAF/MAPK signaling pathways leads to increased metabolism, proliferation, and cell motility [40,41,42,43,44]. It has been shown that mutations in *Ras* and *Raf* oncogenes lead to the constitutive activation of the MAPK signaling pathway, cell differentiation, uncontrolled cell proliferation rates, and cell resistance to apoptosis-induced agents [45,46,47,48]. The liver fluke ESP components might regulate the cell proliferation and motility by activating the EGFR receptor and MAPK signaling pathways, which in turn causes cell cycle progression and cell migration (Figure 7).

*O. felineus* ESP has been shown to contain proteins, vesicles, miRNA, low-molecular-weight metabolites, etc. [3,32]. Our study showed that the EGFR inhibitor abolished the effect of the activation of the proliferation and migration of H69 cells after treatment with the *O. felineus* ESP, which confirms the involvement of this receptor in the activation of the cell signaling cascades. In addition, Marimastat, as an inhibitor of matrix metalloproteases, in particular TACE, also eliminated the effect of the activation of cell proliferation and migration, which can probably be explained by the fact that some EGFR ligands are released by matrix proteases (ADAM17 and TACE) upon the activation of Toll-like receptor 4 (TLR4) [28,29]. Since Marimastat inhibits MMP activity through direct binding, preventing the release of EGFR ligands, we can assume that EGFR activation may occur indirectly, without its direct stimulation by ESP. Furthermore, our previous assessment of the liver transcriptome demonstrated the activation of Toll-like Receptor 4 (TLR4) signaling pathways in opisthorchiasis [3]. Specifically, we observed an increase in the mRNA levels of genes such as *Mmp2* and *Mmp9*, among others involved in the epithelial–mesenchymal transition.

The obtained results are consistent with the effects of other trematodes on the epithelial cells. Specifically, the ESPs of *C. sinensis* and *O. viverrini* have been shown to increase the proliferation and migration of various cell lines [49,50,51,52,53]. Potential active components of the ESP from these trematodes include extracellular vesicles and some active proteins, such as granulin 1, glutathione-S-transferase, etc. The treatment of cells with the ESP of *O. viverrini*, which contains granulin protein (Ov-GRN-1) [16,17], glutathione-S-transferase (OvGST) [54], or isolated extracellular vesicles [55], has been shown to enhance the proliferation and migration rates of various cells, including H69, M213, KKU-100, and KKU-M156 [49,56]. Furthermore, the recombinant *O. viverrini* granulin 1 protein treatment of cells leads to the increased expression of genes related to EGF signaling pathways [56]. Additionally, *O. viverrini* ESP has been reported to induce increased TLR4 mRNA expression in cholangiocytes [51], while *C. sinensis* ESP has been shown to increase TLR4 expression in mouse intrahepatic biliary epithelial cells (MIBECs) [57,58]. Moreover, the activation of the proliferation and migration of hepatocellular carcinoma cells [59] and LO2 cells by *C. sinensis* ESP are mediated by RAS/MAPK/ERK and PI3K/Akt signaling pathways and the activation of EGFR [18]. Increased expression of EGFR and related receptors has been noted in *O. viverrini*-associated CCA [59,60], despite the fact that cholangiocarcinoma is characterized by the weak expression of HER2 and ERbB2 [61].

Additionally, changes in the expression of miRNAs involved in cell proliferation, inflammation, oncogene activation/suppression, migration/invasion/metastasis, and DNA methylation were identified. In particular, the decreased expression of let-7i, a tumor suppressor miRNA, was found to be associated with the ESP-induced upregulation of TLR4 mRNA and protein [62,63]. Increased TLR4 expression was also noted in mouse intrahepatic bile duct epithelial cells (MIBECs) after ESP treatment with *C. sinensis* [58]. Thus, these studies suggest the probable involvement of ESPs in the activation of signaling pathways responsible for pre-carcinogenic and carcinogenic lesions, such as BilIN.

Although a clear relationship between *O. felineus* infection and CCA development has not been established [3], there is some evidence suggesting an increased risk of CCA in individuals infected with *O. felineus* [13]. Cholangiocarcinoma, regardless of its cause, is challenging to diagnose due to its non-specific symptoms and slow-growing tumors that metastasize to distant organs, leading to an unfavorable prognosis for patients [9]. To date, the mechanisms underlying the development of cholangiocarcinoma (CCA) associated with infection by trematodes of the Opisthorchiidae family have not been studied. The involvement of EGFR and MAPK signaling pathways in the development of precancerous changes, including the development of biliary intraepithelial neoplasia and the further development of OF-CCA, provides new insights into biological carcinogenesis and expands our understanding of anticancer therapies. Inhibitors targeting key components of these signaling pathways are potential candidates for anticancer therapy and are already in clinical use [64].

Although our study suggested the involvement of EGFR and MAPK signaling pathways in neoplasia development, this was not shown in detail and needs further research. The precise mechanisms underlying the development of cholangiocarcinoma associated with *Opisthorchis felineus* infection remain insufficiently elucidated. Unfortunately, the specific components of *Opisthorchis felineus* excretory–secretory products (ESPs) responsible for stimulating cell proliferation and migration have not been identified. The mechanisms through which ESPs influence the activation of EGFR and its associated signaling cascades require further investigation. We also were unable to evaluate EGFR expression in patients without cholangiocarcinoma or in conditionally healthy individuals. It remains unclear whether the level of EGFR per cell was increased or whether the number of EGFR-positive cells increased due to the proliferation of a specific cell type.

To address these limitations, we will expand the list of cell lines to study carcinogenesis in primary, non-immortalized cholangiocytes. We also plan to evaluate the role of other cellular signaling pathways in precancerous changes in the bile duct epithelium during *O. felineus* infection. We aim to identify specific components within ESPs that could cause such effects on cholangiocytes.

Nevertheless, we demonstrated the activation of EGFR signaling pathways in the development of biliary intraepithelial neoplasia in opisthorchiasis. However, the role of EGFR signaling pathways in the pathogenesis of biliary intraepithelial neoplasia caused by *Opisthorchis felineus* requires further research.

## 5. Conclusions

In conclusion, a significant increase in EGFR positive staining was observed in both the experimental opisthorchiasis model and patient liver samples. Specific signals for EGFR and EGFRp were detected only in the bile duct epithelium. Moreover, our study showed that the EGFR inhibitor and the matrix metalloproteinase inhibitor eliminated the activation of the proliferation and migration of H69 cells after the treatment with *O. felineus* ESP, which confirms the involvement of these proteins in the activation of the cell signaling cascades.

The activation of EGFR in *O. felineus* infection makes it a promising target for further study in the context of liver-fluke-associated cholangiocarcinoma (CCA) development and for early-stage diagnostics. However, data on potential anti-EGFR therapies for cholangiocarcinoma are limited [65]. Therapeutic agents such as Trastuzumab, a monoclonal anti-HER2 antibody, have shown inhibitory effects on *O. viverrini*-associated CCA cells [66].

Overall, our study unveils novel avenues for exploring the mechanisms of helminth-associated carcinogenesis and for identifying key components of *O. felineus* excretory–secretory products that mediate their mitogenic effects on human epithelial cells.

## Data Availability

All data generated or analyzed during this study are included in this published article.
